# Intakes of calcium, vitamin D, and dairy servings and dental plaque in older Danish adults

**DOI:** 10.1186/1475-2891-12-61

**Published:** 2013-05-16

**Authors:** Amanda RA Adegboye, Lisa B Christensen, Poul Holm-Pedersen, Kirsten Avlund, Barbara J Boucher, Berit L Heitmann

**Affiliations:** 1 Research Unit for Dietary Studies. Institute of Preventive Medicine, Frederiksberg Hospital, Copenhagen DK-2000, Denmark; 2 Department for Community Dentistry. School of Dentistry, Faculty of Health Science, University of Copenhagen, Copenhagen DK-2200, Denmark; 3 Copenhagen Gerontological Oral Health Research Centre. School of Dentistry, Faculty of Health Science, University of Copenhagen, Copenhagen DK-2200, Denmark; 4 Department of Public Health, Section of Social Medicine, and Center for Healthy Aging, University of Copenhagen, Copenhagen DK-1014, Denmark; 5 Danish Aging Research Center, Universities of Aarhus, Southern Denmark and Copenhagen, Aarhus DK-5000, Denmark; 6 Centre for Diabetes, Bart’s & The London School of Medicine & Dentistry, Queen Mary University of London, London E1 2AT, UK

**Keywords:** Calcium, Dairy-foods, Dental plaque, Older adults, Oral-health, Vitamin D

## Abstract

**Background:**

To investigate whether intakes of calcium and dairy-servings within-recommendations were associated with plaque score when allowing for vitamin D intakes.

**Methods:**

In this cross-sectional study, including 606 older Danish adults, total dietary calcium intake (mg/day) was classified as below *vs.* within-recommendations and dairy intake as <3 *vs.* ≥3 servings/ d. Dental plaque, defined as the percentage of tooth surfaces exhibiting plaque, was classified as < median *vs.* ≥median value (9.5%). Analyses were stratified by lower and higher (≥6.8 μg/d) vitamin D intake.

**Findings:**

Intakes of calcium (OR = 0.53; 95% CI = 0.31–0.92) and dairy servings (OR = 0.54; 95% CI = 0.33–0.89) within-recommendations were significantly associated with lower plaque score after adjustments for age, gender, education, intakes of alcohol, sucrose and mineral supplements, smoking, diseases, number of teeth, visits to the dentist, use of dental floss/tooth pick and salivary flow, among those with higher, but not lower, vitamin D intake.

**Conclusion:**

Intakes of calcium dairy-servings within-recommendations were inversely associated with plaque, among those with higher, but not lower, vitamin D intakes. Due to the cross-sectional nature of the study, it is not possible to infer that this association is causal.

## Background

Plaque, a deposit on tooth surfaces consisting of food debris and bacteria, is reduced by good oral hygiene, e.g. brushing and flossing [1]. Long-term plaque may lead to periodontitis and caries [2]. Prevention of these conditions is, therefore, primarily based on plaque control, though dietary factors may also be important [3].

Apart from studies investigating the association between the amount and frequency of free sugars intake and dental caries, little attention has been given to the importance of interactions between dietary nutrients or specific foods on oral health. So far, only few studies have investigated the associations between intake of dairy products (regardless of vitamin D consumption) and oral health [3].

High concentrations of calcium and phosphate in plaque or saliva may reduce bacterial adhesion to enamel and inhibit bacterial biofilm formation [4]. Higher calcium intake may also enhance enamel remineralisation, reduce demineralization [5] and prevent alveolar bone loss [6]. Additionally, casein may protect against caries by reducing bacterial adherence to teeth [7]. Dairy product intakes are particularly relevant as these foods are high in calcium, phosphate, and casein [8]. However, most populations consume substantially less dairy-foods than are recommended for provision of adequate intakes of calcium [9]. Furthermore, vitamin D is essential for calcium absorption [10], and promotes oral health through its effects on bone metabolism and innate immunity [11]. Our previous findings showed associations between tooth loss and calcium intake below recommendations [12] [data not examined for stratification with vitamin D status] and of periodontitis with low dairy calcium intakes with no variation with vitamin D intake [13].

The present study aimed to investigate whether within-recommendation intakes of calcium and dairy-servings are associated with dental plaque score among older adults with higher and lower vitamin D intakes.

## Methods

**Figure 1:**
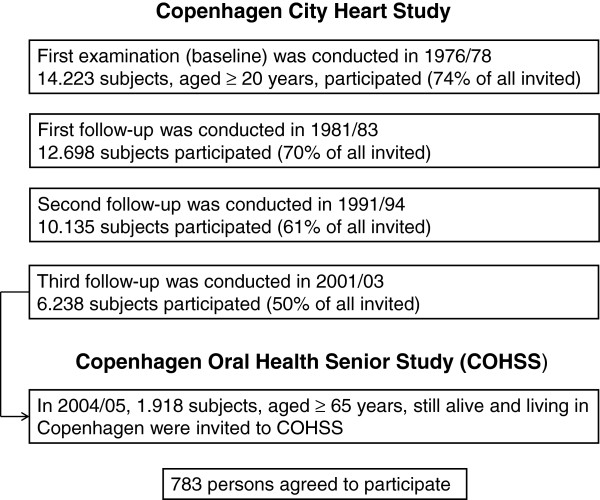
Flow chart for participants in the Copenhagen Oral Health Senior Study.

The Copenhagen Oral Health Senior Study (COHSS) 2004/05, addressed lifestyle factors and oral health in older adults. In total, 1918 of all 6238 subjects, who had participated in the third follow-up of the Copenhagen City Heart Study 2001/03 (detailed published elsewhere [14]), and were still alive, aged ≥65 years old, living in Copenhagen, able to communicate verbally and travel independently, were invited to participate in this oral health study (COHSS), and 783(40%) agreed to participate (Figure 1). Details of participants and non-participants are described elsewhere [15]. Briefly, a larger proportion of non-participants was older and of male gender *vs*. participants (*P* <0.001). COHSS was approved by local Ethical Committees (KF 01-144/01).

### Dietary assessment

Dietary data were collected using a ‘diet history interview’ based on information about diet in the past month, validated for use in older populations [16]. Participants reported on meal contents and patterns at interview through pre-coded questions. Quantitative information on meals, food intakes and portion sizes was collected using photo-series, cups and measures. All interviews were performed by a single dietician.

Total dietary and supplemental intakes^b^ of vitamin D2 + D3 (μg/d) were estimated. Total vitamin D intakes were within-recommendations (15 μg/d for adults aged 51–70 y; 20 μg/d for ≥71 y) [17] in 4% of subjects and intake was, therefore, classified as < median *vs.* ≥median (6.8 μg/d).

Total dietary and supplemental calcium intakes were classified as: below *vs*. within recommendations (1000 and 1200 mg/day for men aged 51–70 and >70 y respectively; 1200 mg/d for women aged ≥ 51 y) [17]. Due to limited information on calcium contents of the supplements used, 800 mg was added to the total amount of calcium intake, but only for those who reported taking calcium supplements daily. Total dairy food intake: <3 *vs*. ≥3 servings/d, as recommended for elders, by the Food Guide Pyramid [18]. Dairy servings refer to one cup of milk, one slice of cheese or one medium yogurt pot.

### Oral examination

One dentist (KH) trained by an experienced clinical examiner (PH-P), performed all oral examinations, including determination of un-stimulated salivary flow rates [19], number of teeth and visible plaque at six sites per tooth, modified from Silness & Löe [20].

Plaque score was determined as % of sites with plaque amongst total sites examined, classified as scoring < median *vs.* ≥median (9.5%).

### Covariates

These included age, gender and primary education (≤7 *vs*. >7 y), current smoking (yes/no) and intakes of alcohol (above *vs*. within-recommendations from the Danish National Board of Health^a^[21]), sucrose (≤10% *vs*. >10% of total energy intake, matching suggested thresholds for increased caries rates and lower vitamin and mineral intakes [22]), and daily mineral supplements (yes/no) over the previous year.

From a questionnaire covering 14 common diseases (e.g. cardiovascular, metabolic, respiratory, hepatic, urinary diseases), current ‘medical condition’ was classified as 0–4 *vs.* ≥5 diseases [15].

Most subjects (98%) reported daily tooth brushing; therefore regular use of dental floss or tooth pick was used for assessing oral hygiene. Elapsed time since last dental-care visit (≤12 *vs*. >12 months), number of teeth, and salivary flow were included in analyses.

### Statistical analysis

Descriptive statistics were presented as ‘mean (±SD)’ or ‘percentage (n)’. Logistic regression was used to assess relationships between calcium and dairy intakes within-recommendations and plaque scores, stratified by vitamin D intakes. Data were analysed with STATA (StataCorp 9.2, TX-USA).

## Results

Of the 783 participants, 96 were edentulous, 78 did not complete the questionnaires, leaving a study population of 606 partial or fully dentate participants with complete information on diet, plaque score and covariates for inclusion in our cross-sectional analyses. Participants did not differ for age, gender, smoking, alcohol, sucrose consumption or medical condition from non-participants. However, non-participants had lower education, used dental floss/tooth pick and visited a dentist less often than participants (data not shown, *P* <0.05).

Total dietary calcium intake ranged from 161 mg to 3439 mg. Dental plaque score ranged from 0% to 90%. Descriptive data are presented in Table 1. Individuals reporting calcium intake below recommendations were older, had higher alcohol consumption, lower vitamin D and dairy-servings intakes and fewer teeth compared to those with adequate calcium intake.

**Table 1:** General characteristics of the study population

	**Calcium intake according to recommendations**			
**Characteristics**	**Total % (n)**	**Below % (n)**	**Within % (n)**	***P***
Age*	75.5 (6.4)	75.9 (6.3)	74.4 (6.6)	0.01
≤ 7 y of education	30.7 (186)	32.1 (144)	26.6 (42)	0.20
Males	39.4 (239)	36.2 (162)	48.7 (77)	0.005
Smoking	22.4 (136)	23.4 (106)	19 (30)	0.226
Vitamin D intake < 6.8 μg/d	50.7 (307)	52.9 (237)	44.3 (70)	0.063
Vitamin D intake (μg/d )*	7.4 (4.9)	7.1 (4.8)	8.2 (5.1)	0.02
Calcium intake (mg/d)*	989 (460)	776 (211)	1591 (439)	< 0.0001
Dairy intake < 3 servings/d	62.4 (378)	80.6 (361)	10.8 (17)	< 0.001
> 10% energy from sucrose	16.2 (98)	15.4 (69)	18.3 (29)	0.386
Alcohol > recommendations	28.7 (174)	31 (139)	22.1 (35)	0.048
Current diseases ≥ 5	52 (315)	51.6 (231)	53.2 (84)	0.729
> 12 months since last dental care visit	8.7 (53)	9.1 (41)	7.6 (12)	0.551
No regular use of dental floss/tooth pick	59 (357)	60.9 (273)	53.2 (84)	0.09
Number of teeth*	20.2 (7.8)	19.8 (7.9)	21.3 (7.4)	0.03
Plaque Score (%)*	14.4 (20)	14.9 (21)	13.2 (19)	0.370

Data presented as % (n) unless otherwise stated. * Data presented as mean (SD).

**Figure 2:**
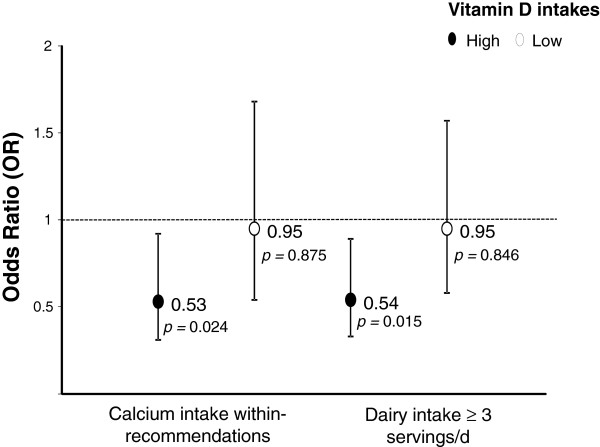
**Association between calcium and dairy servings intakes and plaque score, stratified by vitamin D intakes.** Models adjusted for age, gender, education, intakes of alcohol, sucrose and mineral supplements, smoking, current diseases, number of teeth, visits to the dentist, use of dental floss/tooth pick and salivary flow. Plaque score classified as < *vs.* ≥ 9.5%. Analyses were stratified by lower and higher (≥6.8 μg/d) vitamin D intake.

Table 2 shows the overall associations of calcium, dairy products and vitamin D intakes with plaque score. The results showed protective effects of calcium, dairy products and vitamin D intakes on plaque score, but none of the associations were statistically significant. When the analysis were stratified by vitamin D intake, it was observed that increased intakes of calcium and dairy-servings (within-recommendations) were significantly associated with lower plaque score, amongst those with higher vitamin D intakes, after adjustment for age, gender, education, intakes of alcohol, sucrose and mineral supplements, smoking, current diseases, number of teeth, visits to the dentist, use of dental floss/tooth pick and salivary flow. Associations were not significant amongst those with lower vitamin D intakes (Figure 2).

**Table 2:** Overall associations of calcium, dairy products and vitamin D with plaque score

**Calcium**		**Within recommendations**	**Below recommendation**	***P***
	n	158	448	
	OR (95% CI)	0.72 (0.49-1.0)	1.0	0.090
**Dairy products**		≥ 3 servings/day	< 3 servings/day	*P*
	n	228	378	
	OR (95% CI)	0.72 (0.51-1.0)	1.0	0.057
**Vitamin D**		≥ median	< median	*P*
	n	299	307	
	OR (95% CI)	0.96 (0.68-1.35)	1.0	0.083

Models adjusted for age, gender, education, intakes of alcohol, sucrose and mineral supplements, smoking, current diseases, number of teeth, visits to the dentist, use of dental floss/tooth pick and salivary flow.

Plaque score classified as < *vs.* ≥ 9.5%.

Additional subgroup analyses by gender, age, alcohol consumption and number of teeth were performed, but no significant difference between groups were found.

## Discussion

Our findings show that higher intakes of calcium and dairy-servings (within-recommendations) are associated with lower plaque scores, when vitamin D intakes are ≥6.8 μg/d, but not when intakes are <6.8 μg/d.

To our knowledge, this is the first observational study examining associations between calcium and dairy-servings intakes and plaque score. However, consistent evidence suggests that higher intakes of calcium, dairy-foods and vitamin D are inversely associated with caries and periodontitis [8,11,23], though earlier studies on dairy-foods and oral health have not stratified analyses by vitamin D intake.

Dietary calcium absorption is critical for calcium availability for bone mineralization [17] and vitamin D status is an important factor influencing intestinal calcium absorption [24]. Our data, therefore, suggests that better vitamin D intakes facilitate beneficial effects of higher calcium intakes, probably by enhancement of calcium absorption.

Weaknesses of this study include the high drop-out rate from the original cohort and exclusions due to insufficient data availability. However, participants and non-participants showed no significant differences in major socio-demographic and lifestyle characteristics. Lower risks in participants would have led to underestimations rather than overestimates of the reported associations. Another limitation was the lack of detailed data for supplemental calcium intakes. However, underestimates of total calcium intake would have attenuated the significance of our findings.

Silness & Löe’s method for plaque assessment [20] focusses on plaque quantity at time of inspection and does not reveal how long plaque has been present. In considering plaque as an etiological risk-factor for caries and periodontitis, time must be a most important factor [2]. Human responses and individual susceptibility to plaque vary with gender, ethnicity, medical status, genotype and manual dexterity for achieving a satisfactory level of oral cleanliness through self-care [1]. Therefore, individuals with similar hygiene practices may present different plaque scores. In this study, higher intakes of calcium and dairy-foods were associated with a lower plaque score, even after adjustment for many socio-demographic factors, health conditions, lifestyle and oral hygiene, influencing plaque formation.

Kappa for intra-examiner plaque assessment was not calculated in this study. However, for caries assessment the Kappa-value for the intra-observer reliability was 0.97, which may be considered as excellent agreement. Since visual inspection of detectable plaque is a much simpler procedure for an experienced dentist, it is reasonable to assume that the present study was unlikely to have been significantly affected by intra-observer variation.

Finally, most participants reported daily tooth brushing, but it was not possible to distinguish between tooth brushing once or twice a day.

## Conclusion

Our cross-sectional data corroborate the hypothesis that calcium and dairy-foods intakes within-recommendations are associated with lower plaque scores [23] amongst those with higher, but not lower, vitamin D intakes. However, it is not possible to infer that these associations are causal. Given the high percentage of individuals with below-recommendation intakes of calcium and vitamin D, consumption of foods rich in calcium and providing vitamin D at 6.8 μg/day or more should be promoted, as it may contribute to reduction of plaque formation and protection of oral health, whilst awaiting data from further studies on the effects of intakes of vitamin D currently advised by the Institute of Medicine [17] or above, on dental health.

### Endnotes

^a^ The Danish National Board of Health recommends a maximum consumption of 168 g alcohol/wk for women and 252 g alcohol/wk for men.

^b^ For daily supplemental vitamin D, multivitamins, or cod-liver oil, intakes of 10 μg, 5 μg or 6 μg of vitamin D were added, respectively.

## Abbreviations

COHSS: Copenhagen Oral Health Senior Study; CI: Confidence interval; d: Day; OR: Odds ratio; SD: Standard deviation; vs: Versus; wk: Week; y: Years.

## Competing interests

The authors declare that they have no competing interests.

## Authors’ contributions

ARA carried out the statistical analysis and did most of the writing. BJB contributed to plans for data analysis. PH-P and KA designed and supervised the study. All authors helped draft the manuscript and read and approved the final manuscript.
